# Survey of biochemical and oxidative profile in donkey foals suckled with one natural and one semi-artificial technique

**DOI:** 10.1371/journal.pone.0198774

**Published:** 2018-06-06

**Authors:** Pasquale De Palo, Aristide Maggiolino, Marzia Albenzio, Elisabetta Casalino, Gianluca Neglia, Gerardo Centoducati, Alessandra Tateo

**Affiliations:** 1 Department of Veterinary Medicine, University “A. Moro” of Bari, Valenzano, Bari, Italy; 2 Department of Agricultural Food and Environmental Sciences, University of Foggia, Foggia, Italy; 3 Department of Veterinary Medicine and Animal Production, Federico II University, Naples, Italy; 4 Department of Soil, Plant and Food Science, University of Bari, Bari, Italy; University of Illinois, UNITED STATES

## Abstract

Dairy donkey milking procedures require separating foals from their dams for a few hours a day. Artificial suckling in this species is a good technique for improving milk production and foal welfare. The aim of the work is to compare the effect of two different diets on donkey foals when separated from jennies for milking procedures with and without a milk replacer. Forty newborn Martina Franca donkey foals were subdivided into two experimental groups. Both groups were separated from their respective dams from 8.00to 20.00to allow the jennies to be milked. During the separation, all the foals had access *ad libitum* to water, hay and feed. During the separation period, one group had the availability of a mechanical milk replacer dispenser, so foals were partially artificially suckled (AS), while the other group had no milk replacer available, and so were totally naturally suckled (NS). The AS group had milk replacer availability until 120±7d of life. Both groups were naturally weaned at 168±7d. Blood samples were collected weekly starting from birth until two wks after weaning (i.e. at 182d), from all the foals included in the trial. Almost all the analytes were influenced by suckling technique and age of foals. Alanine-aminotransferase, aspartate-aminotransferase, alkaline phosphatase, NEFA, lipid hydroperoxides, serum proteins showed the greatest differences between the two experimental groups. Separating foals from their dams for 12hdaily for 24 weeks does not lead to pathological subclinical and metabolic conditions, thus confirming the high rusticity and resistance of the donkey.

## Introduction

In recent years, donkey breeding has started to become the focus of interest in Western countries [1]. Greater attention is being paid to equid milk, from both donkey and horse [2, 3] as functional foods for treating children suffering from allergies or intolerance to cow milk, raising interestin these species and their products [4]. Despite the rise in interest, there are some animal production-related problems due to the lack of scientific research aimed at improving production in this species.

In milking jennies, sharing milk with foals was one of the main problems. The traditional technique involves a fasting period for foals in order to milk their mothers, particularly during their first days of life when they are unable to eat enough solid feed to satisfy their energy requirements [5]. In fact, this practice leads foals to intake water, hay and concentrate before they can take and digest it in adequate quantities for their needs in the first months of life, resulting in an alteration of natural suckling that could even induce some alterations in animal welfare and physiology [1]. It is known that the equid udder has a lower storage capability compared with ruminants and is more adapted to frequent milk removal [6]. This aspect is strictly linked to the high suckling frequency of foals if compared to other domestic ruminants [7]. The introduction of an innovative technique for partially suckling foals with a milk replacer could provide a solution [5]. Allowing foals to suck milk even when they are away from their dams could improve metabolic status and welfare levels in donkey foals. This paper therefore investigates these aspects in order to ensure better welfare, health status and productivity for donkey foals, without losing the jennies’ milk production. The aim of this study was to evaluate biochemical and oxidative parameters in sucking donkey foals, comparing two different breeding techniques during the daily separation from the jennies for milking procedures: one group without milk available, fed only with solid food and the second group with a continuous milk availability, using a milk replacer together with solid food, during the first 6 months of life, in order to assess any differences in metabolic profile between the two foal groups.

## Materials and methods

This study was approved by the Ethics Committee for animal testing–CESA (process number 58337-X/10).

### Animals and feeding management

**Fig 1:**
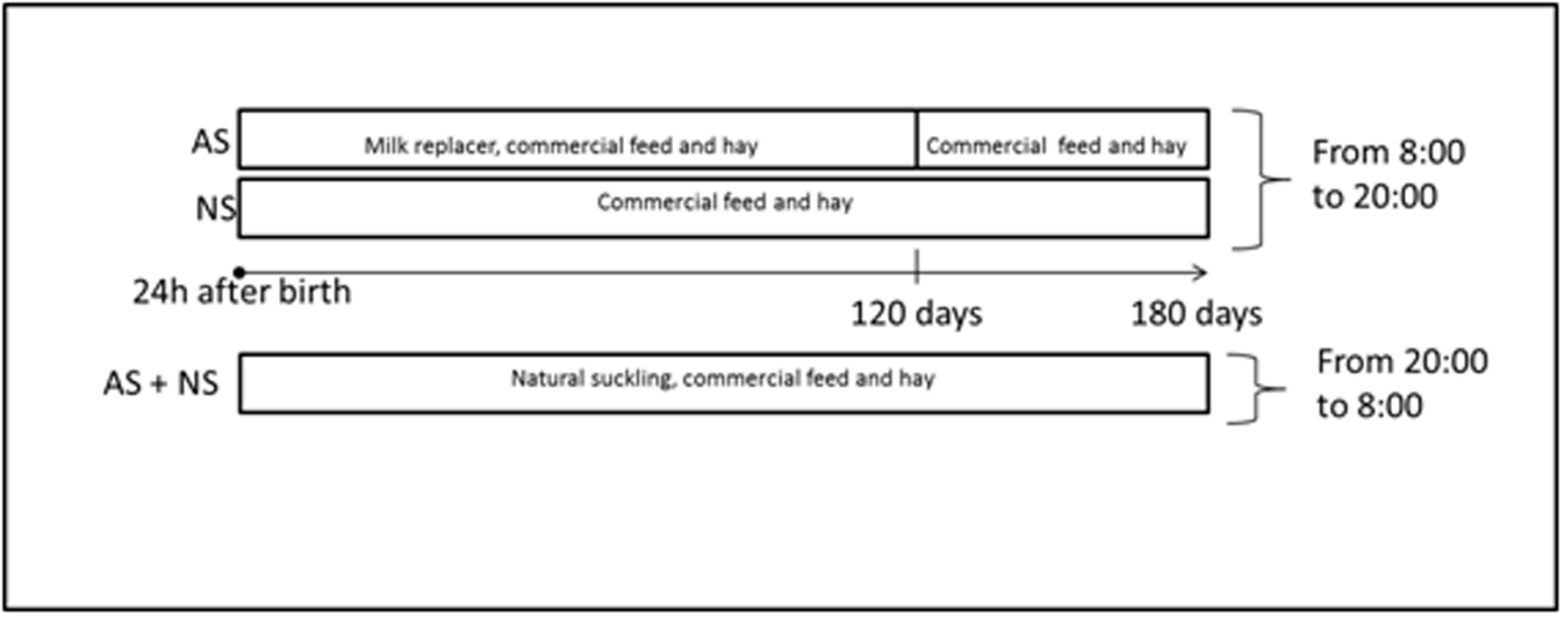
Experimental design.

Forty Martina Franca jennies and their foals, born within a time range of 35 days on the same farm were included in the trial. The sample size was calculated starting from the total Martina Franca breed population (about 700 heads), setting alpha to 0.05, and beta to 0.20 (which allows for 80% power), using mean values and standard errors of the item that showed the highest variability between biochemical parameters in donkeys from literature, [8] that was blood Alanine aminotransferase (ALT). The minimum detectable contrast between the two experimental groups of the same age was set at 2.5 UI/L. Immediately after birth, foals naturally suckled colostrum from their dams. Foals were randomly assigned to two groups (Fig 1). A basic randomization method was used on the jennies before foaling, using the Microsoft Excel “RANDBETWEEN” function as a random number generator, with the following rules: from 1 to 5: AS group; from 6 to 10: NS group. Twenty foals were partially artificially-suckled (AS), while the other 20 foals were completely naturally suckled (NS). From 8.00to 20.00, both groups were separated from their mothers in order to allow the jennies to be milked (average daily milk yield per jenny: 1.73 kg). By contrast, at night both were reared with their dams, allowing natural suckling.

During daytime separation from the jenny, both groups were kept in two indoor stalls allowing 8m^2^ per animal. During the night, the two groups of foals stayed with their dams, but in two different indoor stalls, with a space allowance of 12 m^2^ per jenny-foal pair.

Each stall used in the trial was equipped with four individual water troughs with floats, available both for foals and jennies, daily cleaned, racks for hay, and fodder troughs for concentrates.

**Fig 2:**
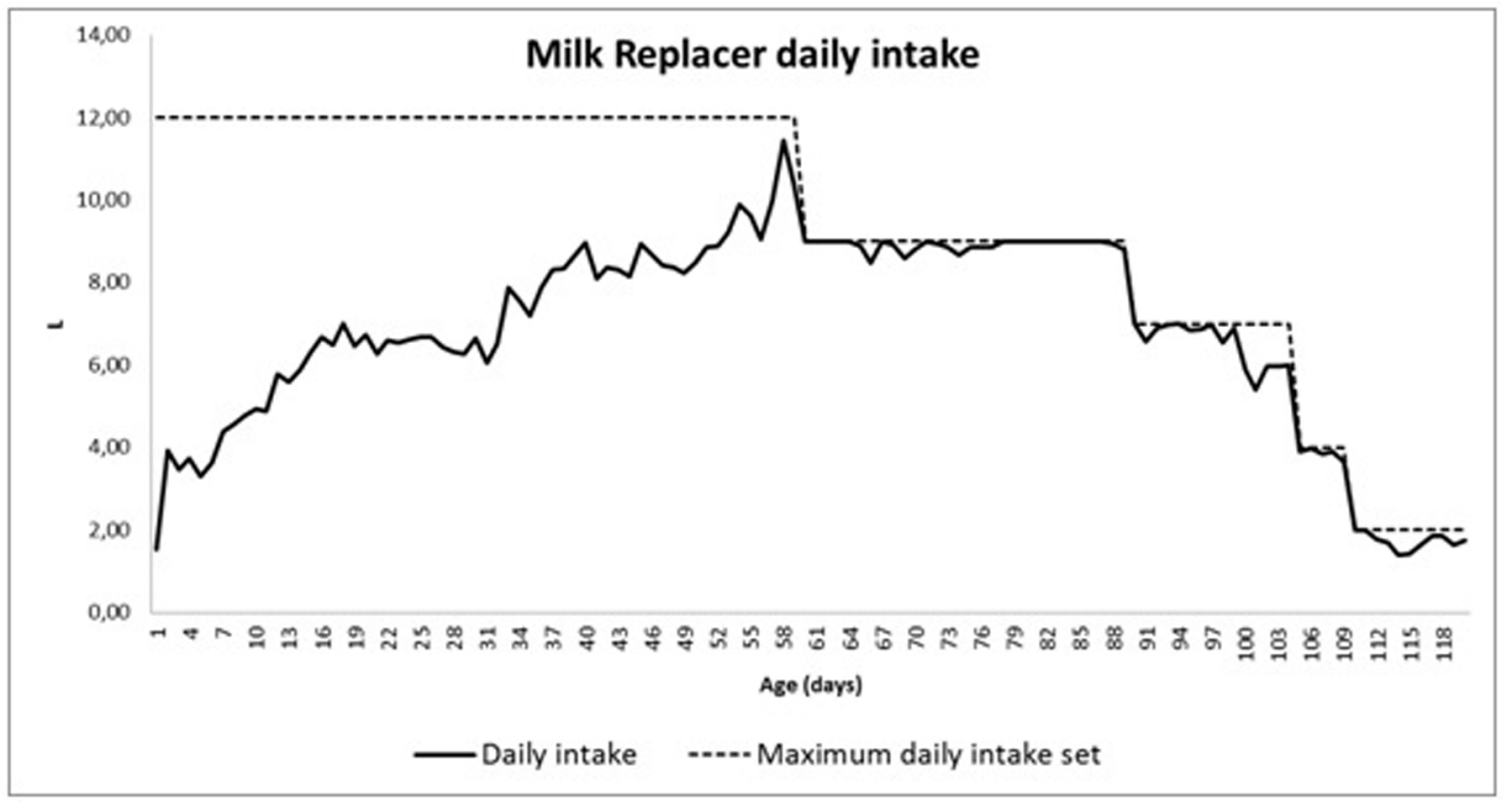
Maximum amount of daily available artificial milk (dashed line) and mean milk intake of donkey foals during 120 days of artificially suckling.

In all the stalls where foals were kept, with or without dams, *ad libitum* fresh water, foal starter feed (2% DM of live body weight) and oat hay (*ad libitum*), satisfying nutritional requirements on the basis of the estimated dry matter intake [9]. Only the AS group stall was equipped with an automatic calf-suckling machine with two automatic milk feeders (Urban GmbH & Co.KG, Wüsting, Germany). Each station and its teats were modified [7]. The composition and reconstitution of milk replacer and the technique for adapting donkey foals to suck from the milking machine were as previously described [9]. The AS group had milk replacer available during the daytime when they were separated from their dams until 120±7 days (16±1 weeks) of life, while the NS group only had water and solid feed at their disposal. Each foal in the AS group was equipped with a transponder fixed to a lightweight collar round its neck, which was used by the automatic suckling machine to recognize individual animals. The machine was settable for total daily amount of milk replacer available per foal, and consequently it was subdivided into a total amount per hour. The maximum amount of milk replacer available was decreased with increasing foal age (Fig 2). Foals in the AS group showed a significantly higher growth rate than those in the NS group from birth to 6 months of life (0.51 kg/d vs 0.43 kg/d, respectively) [5].

### Milk replacer ingestion

AS foals started suckling at 24h of life, on less than 2L of milk replacer, being hand-fed with a bottle equipped with a teat, until the foals associated the automatic milk feeders with suckling. The first donkey learnt to drink from the suckling station within 48h, the second and the third within the next 24h, and the remainder within another 15–18 hours. Throughout the experimental period, we detected no signs of sickness, through weekly veterinary clinical examination procedures, consisting in the general appearance observation, heart auscultation, rectal temperature recording, inspection of oral and ocular mucosae, capillary refill time and skin tent test for the evaluation of hydration status.

### Blood sampling and analysis

Blood samples were aseptically obtained via jugular vein puncture using disposable needles (23G), with a negative pressure system for serum (9mL tubes without anticoagulant) and plasma (9mL tubes with 15 USP U/mL of heparin) (Becton, Dickinson Canada Inc, Vacutainer^®^, Oakville, Canada). Heparinized tubes where immediately centrifuged (1500 g for 10 min) whilst tubes without anticoagulant were allowed to clot at room temperature prior to being centrifuged. All samples were transported in a cooler with ice to the Animal Science Section Laboratory at the Veterinary Medicine Department of Bari (Italy)for immediate processing, with a mean interval of 13h between blood collection and laboratory analysis.

Blood samples were collected weekly, from birth until two weeks after weaning from both experimental groups. Blood was collected throughout the trial period at the same time of day (from 8pm to 9pm), soon before merging foals with their dams.

Clinical biochemistry parameters were obtained from the serum samples using an automated biochemistry analyzer (CS-300B;Dirui, Changchun, China). The following parameters were assessed: alanineaminotransferase (ALT), aspartate aminotransferase (AST), creatinephosphokinase (CPK), lactate dehydrogenase (LDH), alkaline phosphatase (ALP), glucose (Glu), blood urea nitrogen (BUN), creatinine (Crea), total serum protein (Tot Prot), albumin (Alb), cholesterol (Chol), triglycerides (Trig), non-esterified fatty acids (NEFA), calcium (Ca), phosphate(P), magnesium (Mg), chloride (Cl), (Gesan Production Kit, Campobello di Mazara, Trapani, Italy). Besides, Globulins (Glob) and Albumin/Globulin ratio (Alb/Glob) were calculated starting from Total Protein and Albumin parameters. Before beginning each analytical session, we used the standards furnished in the assay kits to calibrate the multi-parameter analyzer (Seracal, Gesan Production Kit, Campobello di Mazara, Trapani, Italy). After setting the calibration curve, two multi-parameter control sera (Seracontrol N and Seracontrol P, Gesan Production Kit, Campobello di Mazara, Trapani, Italy) were used to verify internal accuracy, considered satisfactory when the measured value deviated by no more than 3.00% from the manufacturer’s declared values. Each sample was analyzed in triplicate, and the value used for the data set was the mean of the three recordings for each item analyzed.

Plasma samples were used for oxidation parameters: thiobarbituric acid reactive substances (TBARS), lipid hydroperoxides (Hy) and protein carbonyl (PC) levels. Thiobarbituric acid reactive substances were determined spectrophotometrically [10], Hywere measured spectrophotometrically by an iodometric method; PC levels were determined spectrophotometrically according to [11].

Annex 2 lists the Intra- and Inter-assay variation coefficients for each parameter.

### Statistical analysis

All data records were tested for normality with the Shapiro-Wilk test from the UNIVARIATE procedure of SAS [12] and results indicated that all data, and their residuals were distributed normally [Shapiro-Wilk test (W) ≥ 0.90].

Best linear unbiased estimation (BLUE) was used to estimate fixed effects. To derive BLUE of the fixed effects, a mixed model, considering the week effect as repeated measurements, was used, by the MIXED procedure of SAS [12]. We applied mixed ANOVA because this model compares the mean differences between groups that have been split into two "factors" or, in our case, into two different suckling techniques. Age and suckling techniques were used as fixed effects and individual foals as random effects. To study the effects of age and suckling technique, and their binary interactions on hematobiochemical items, the following mixed linear model was used:

$${\text{Y}}_{\text{ijk}}=\mu +{\text{A}}_{\text{i}}+{\text{S}}_{\text{j}}+{\left(\text{A}\;\times \;\text{S}\right)}_{\text{ij}}+{\text{e}}_{\text{ijk}},$$

in which Y_ijk_ is the observed value of the dependent hematobiochemical variable determined from a sample taken from each animal, μ is the overall mean, A_i_ is the fixed effect of the i^th^age, expressed in weeks (i = 1:26), S_j_ is the fixed effect of the j^th^ suckling technique (j = 1:2), (A × S)_ij_ is the first-order binary interaction between the i^th^ age and the j^th^ suckling technique, and e_ijk_ is the residual error.

The binary interaction between Age and Suckling technique did not affect the hematobiochemical parameters (P > 0,25), so it was deleted by the final model.

Table 1 shows the P values obtained using the statistical model.

**Table 1:** Analysis of variance.

Parameter^1^	Age	Suckling technique	Suckling technique × Age
Total protein	< .0001	< .0001	< .0001
Albumin	< .0001	< .0001	< .0001
Urea	< .0001	0.2724	< .0001
Creatinine	< .0001	0.0058	0.0004
Glucose	< .0001	0.0450	0.0002
NEFA	< .0001	0.0002	0.0130
Triglyceride	< .0001	< .0001	< .0001
ALT	0.0030	<0.0001	0.5652
AST	0.0669	< .0001	0.0255
ALP	0.7544	< .0001	0.4964
LDH	< .0001	0.0283	< .0001
CK	0.0030	0.0028	0.0066
Cholesterol	< .0001	< .0001	0.0174
Calcium	< .0001	0.0258	< .0001
Phosphorus	< .0001	0.5930	< .0001
Magnesium	< .0001	< .0001	0.0537
Chlorine	0.0090	0.0037	0.0606
TBARs	0.0001	0.9519	0.0067
Lipid hydroperoxides	0.0002	< .0001	0.0250
Protein carbonyls	< .0001	< .0001	< .0001

^1^ALT = alanine aminotransferase; AST = aspartate aminotransferase; ALP = alkaline phosphatase; LDH = lactate dehydrogenase; CPK = creatinine phosphokinase; TBARs: Thiobarbituric acid reactive substances.

The *post hoc* Tukey’s test was applied for repeated measures on each suckling technique in order to evaluate differences over time. Significance was set as P< 0.05. All data were expressed as least-squares means and SD.

## Results

**Fig 3:**
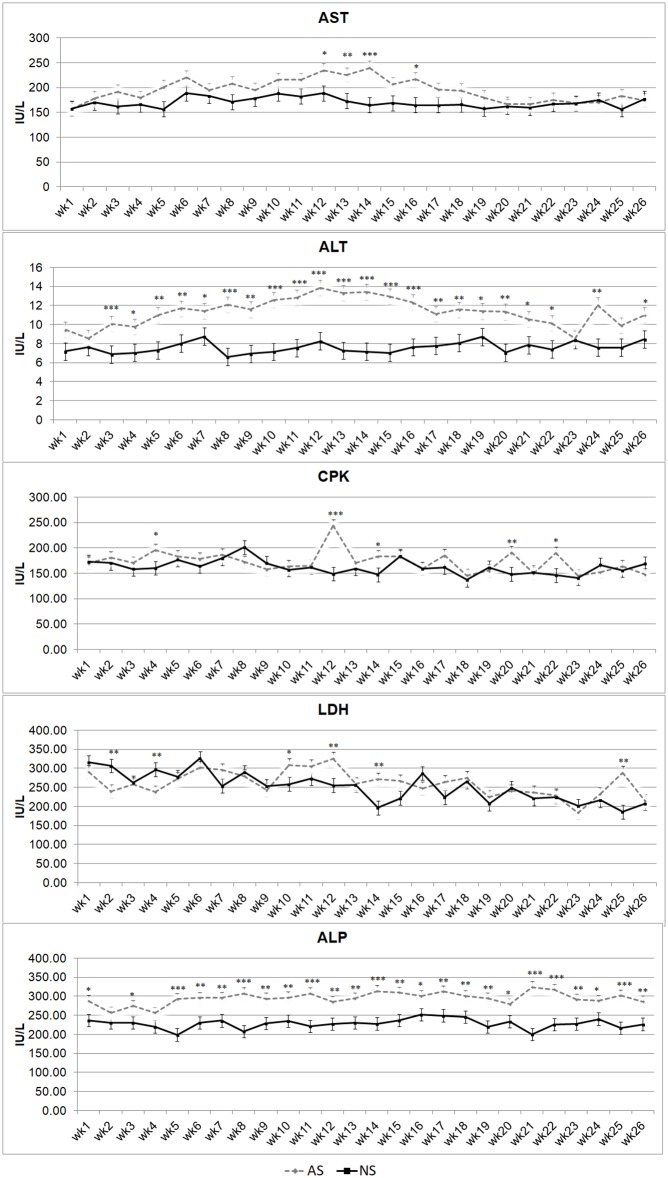
Alanine aminotransferase (ALT), aspartate aminotransferase (AST), creatine phosphokinase (CPK), lactate dehydrogenase (LDH) and alkaline phosphatase (ALP) serum concentrations in artificially-suckled(AS) and naturally-suckled (NS) donkey foals during the first 26 weeks of life. (Values are Least Square Means; bars represents Standard Error of the Mean). Statistical differences between the two groups at the same week were expressed: * = P < 0.05; ** = P < 0.01; *** = P < 0.001.

Fig 3 shows trends for AST, ALT, CPK, LDH, ALP. The AS group showed higher AST and ALT concentrations than the NS group throughout the trial period, although they were statistically significant only between weeks 12 and 16 of life for AST and from wk1 to 26 for ALT. Creatine phosphokinase concentration trends in AS and NS foals are superimposable, although the AS group showed higher concentrations in weeks 4, 12, 14, 20 and 22 (P < 0.01). Lactate dehydrogenase showed differences between the AS and NS groups in weeks2, 4, 10, 12, 14 and 25 (P < 0.01). Similarly to ALT, ALP concentration also showed great differences between the experimental groups, with higher levels throughout the experimental period for AS than for NS, with high statistical significance levels except for weeks 2 and 4.

**Fig 4:**
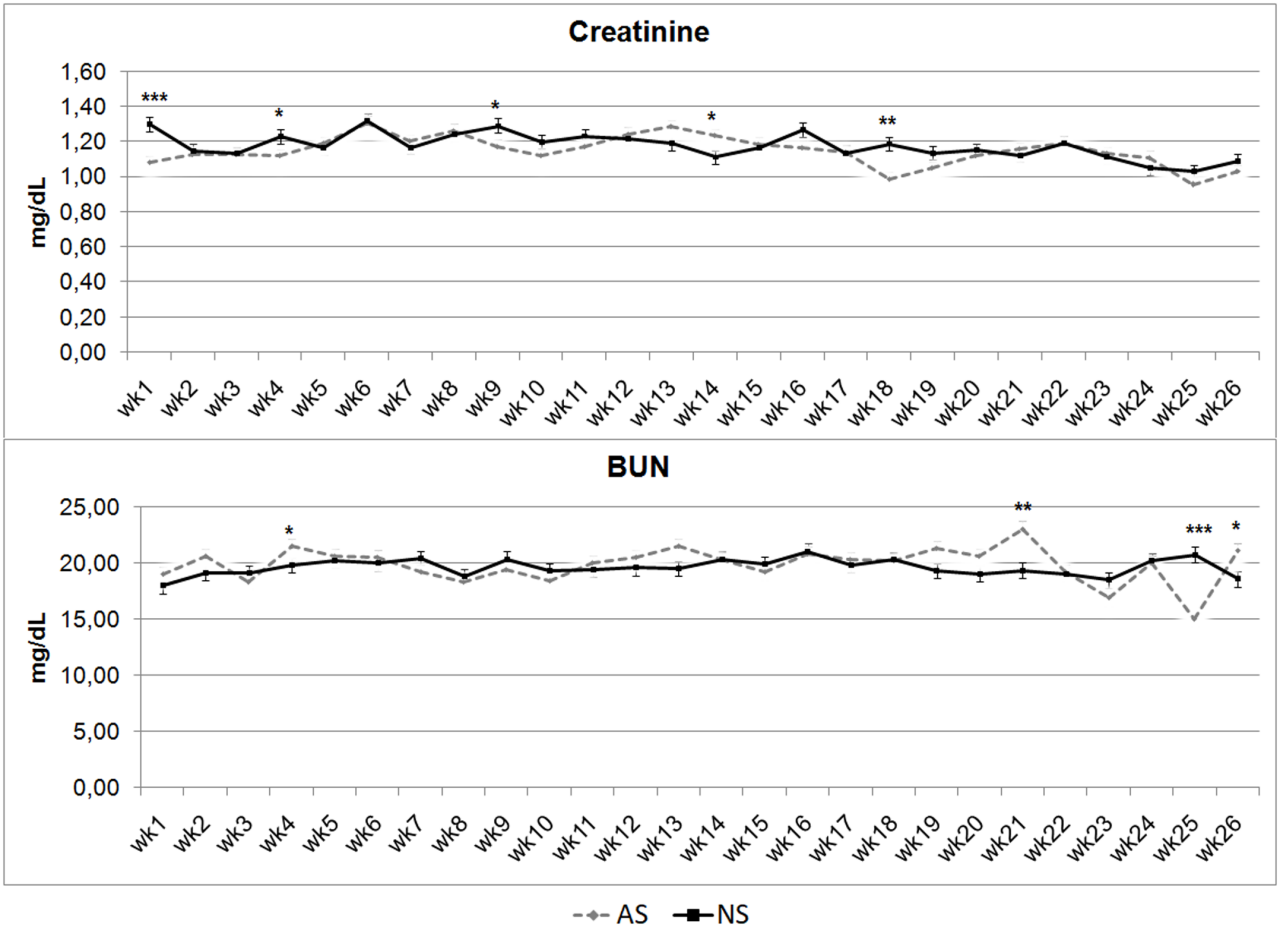
Creatinine and blood urea nitrogen (BUN) serum concentrations in artificially-suckled (AS) and naturally-suckled (NS) donkey foals during the first 26 weeks of life. (Values are Least Square Means; bars represents Standard Error of the Mean). Statistical differences between the two groups at the same week were expressed: * = P < 0.05; ** = P < 0.01; *** = P < 0.001.

Fig 4 shows trends for Crea and BUN concentrations, showing almost identical trends in the AS and NS groups. Creatinine showed higher concentrations in NS foals than in AS ones in wks 1 (P < 0.001), 4, 9 (P < 0.05), and 18 (P < 0.01), while wk 14 revealed an inversion of concentrations, with higher values for Crea in AS than in NS (P < 0.05).

Blood Urea Nitrogen had higher concentration values in wks 2, 26 (P < 0.05) and21 (P < 0.01) in AS, and at wk 25 in NS (P < 0.001).

**Fig 5:**
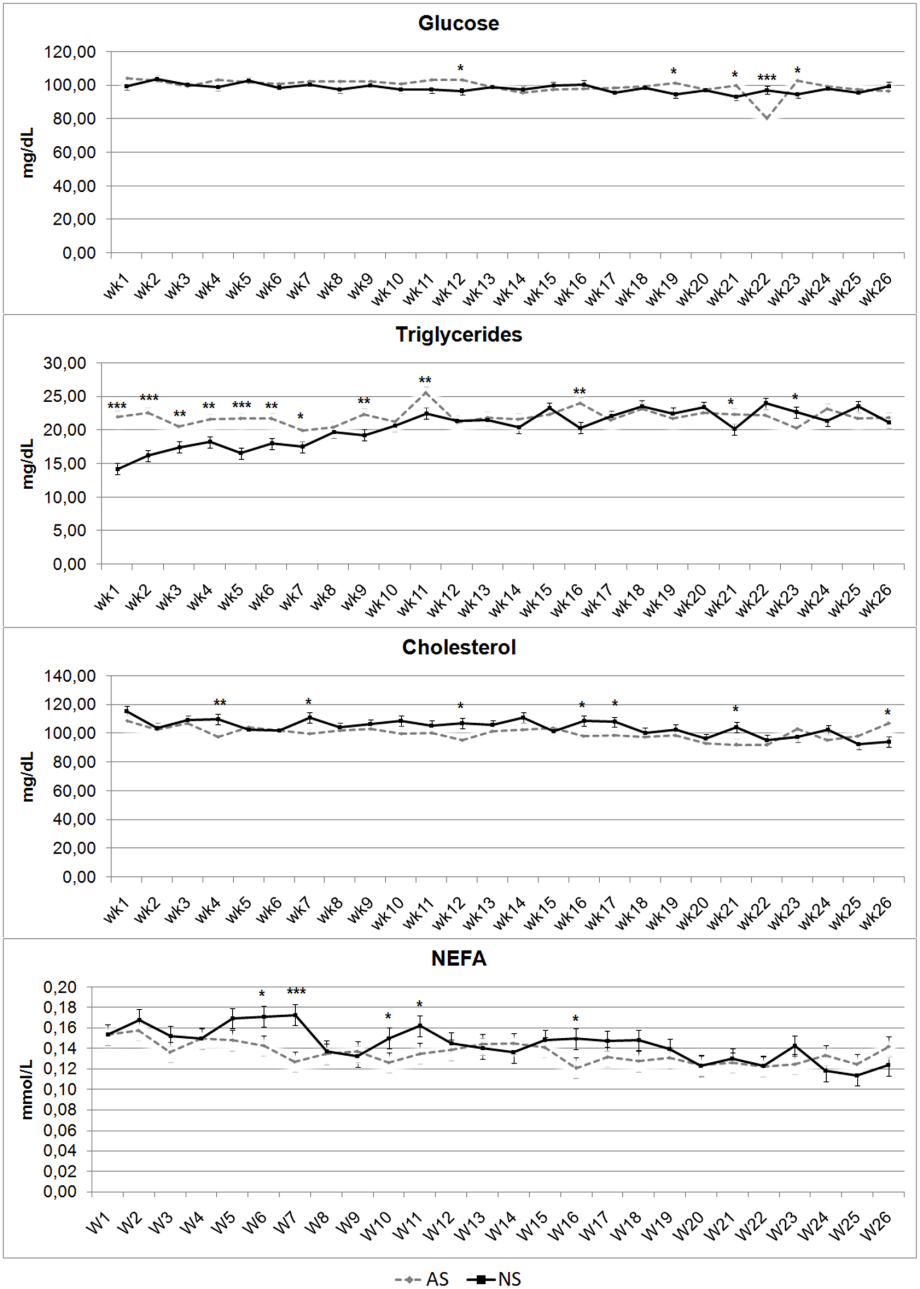
Glucose, triglycerides, cholesterol and non-esterified fatty acids (NEFA) serum concentrations in artificially-suckled (AS) and naturally-suckled (NS) donkey foals during the first 26 weeks of life.(Values are Least Square Means; bars represents Standard Error of the Mean). Statistical differences between the two groups at the same week were expressed: * = P < 0.05; ** = P < 0.01; *** = P < 0.001.

Fig 5 shows trends for Trig, Chol, NEFA and Glu concentrations. Triglyceride levels were higher in AS foals in wks 1, 2, 5 (P < 0.001), 3, 4, 6, 9, 11 (P < 0.01), 7, 16 and 21 (P < 0.05). Cholesterol values were higher in NS throughout, and in particular in wks 4 (P < 0.01), 7, 12, 16, 17, 21 (P < 0.05). At wk 26, Chol concentrations showed an inversion, with higher levels in AS than in NS (P < 0.05). Non-esterified fatty acids almost always showed higher levels in NS foals, with significant differences in wks 6, 10, 11, 16 (P < 0.05) and7 (P < 0.001). Higher glucose concentrations were recorded in AS foals at wks 12, 19, 21 (P < 0.05) and 23 (P < 0.01). At week 22, NS foals had higher Glu concentrations than AS (P < 0.001).

**Fig 6:**
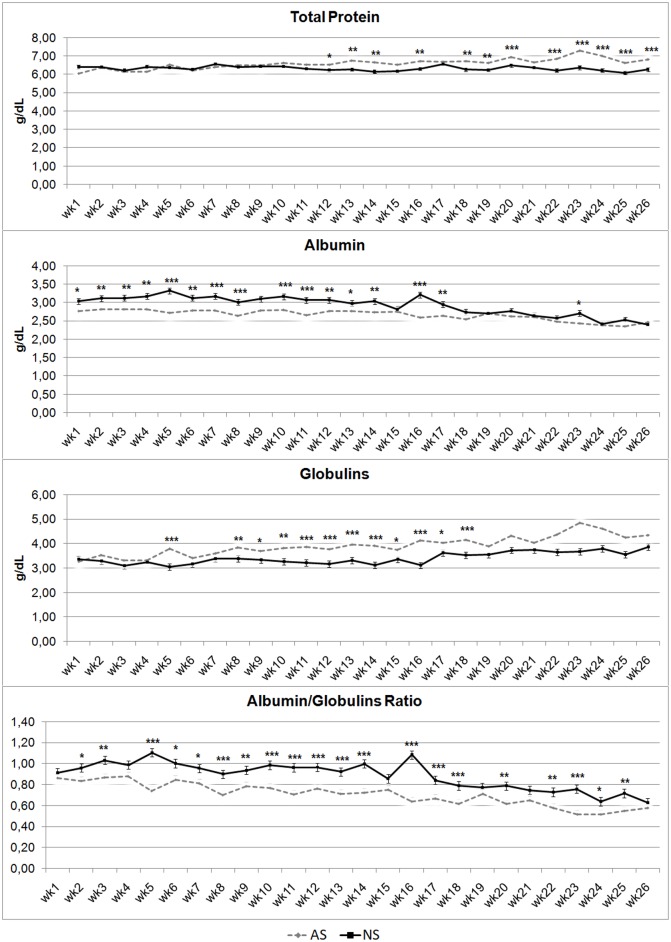
Total protein, albumin, globulins and albumin/globulin ratio levels in artificially-suckled (AS) and naturally-suckled (NS) donkey foals during the first 26 weeks of life. (Values are Least Square Means; bars represents Standard Error of the Mean). Statistical differences between the two groups at the same week were expressed: * = P < 0.05; ** = P < 0.01; *** = P < 0.001.

Fig 6 shows trends for blood serum protein concentrations. Total proteins had a superimposable trend in NS and AS until wk 12 (P > 0.05), when the TP values in AS foals tend to increase compared with NS ones (P values ranging from < 0.05 to < 0.001). Albumin tended to have an opposite trend to TP, with greater differences in the first two thirds of the investigation period, followed by an overlap of Alb from wks 18 to 26, with the exception of wk 23,where Alb values in NS were higher than in AS foals (P < 0.05). Albumin showed higher concentrations in NS foals than in AS ones throughout, although with different levels of statistical significance, ranging from P < 0.05 (wks 1 and 13) to P < 0.001 (wks 5, 7, 8, 10, 11,16). Globulin showed an opposite trend to Alb, with higher levels in AS foals than NS. Besides, these differences are highly statistically significant (P <0.001 in wks 5, 11 to 13, 14,16, 18, 20, 22 to 25) throughout almost the entire investigation period, except for wks 1 to 4, 6, 7 and 21. Albumin/Globulin ratio showed higher levels in NS foals throughout than in AS (P < 0.001 in wks 5, 8, 10 to 14,16, 17, 18, 23; P < 0.01 in wks 3, 9, 20, 22, 25; P < 0.05 in wks 2, 6, 7 and 24).

**Fig 7:**
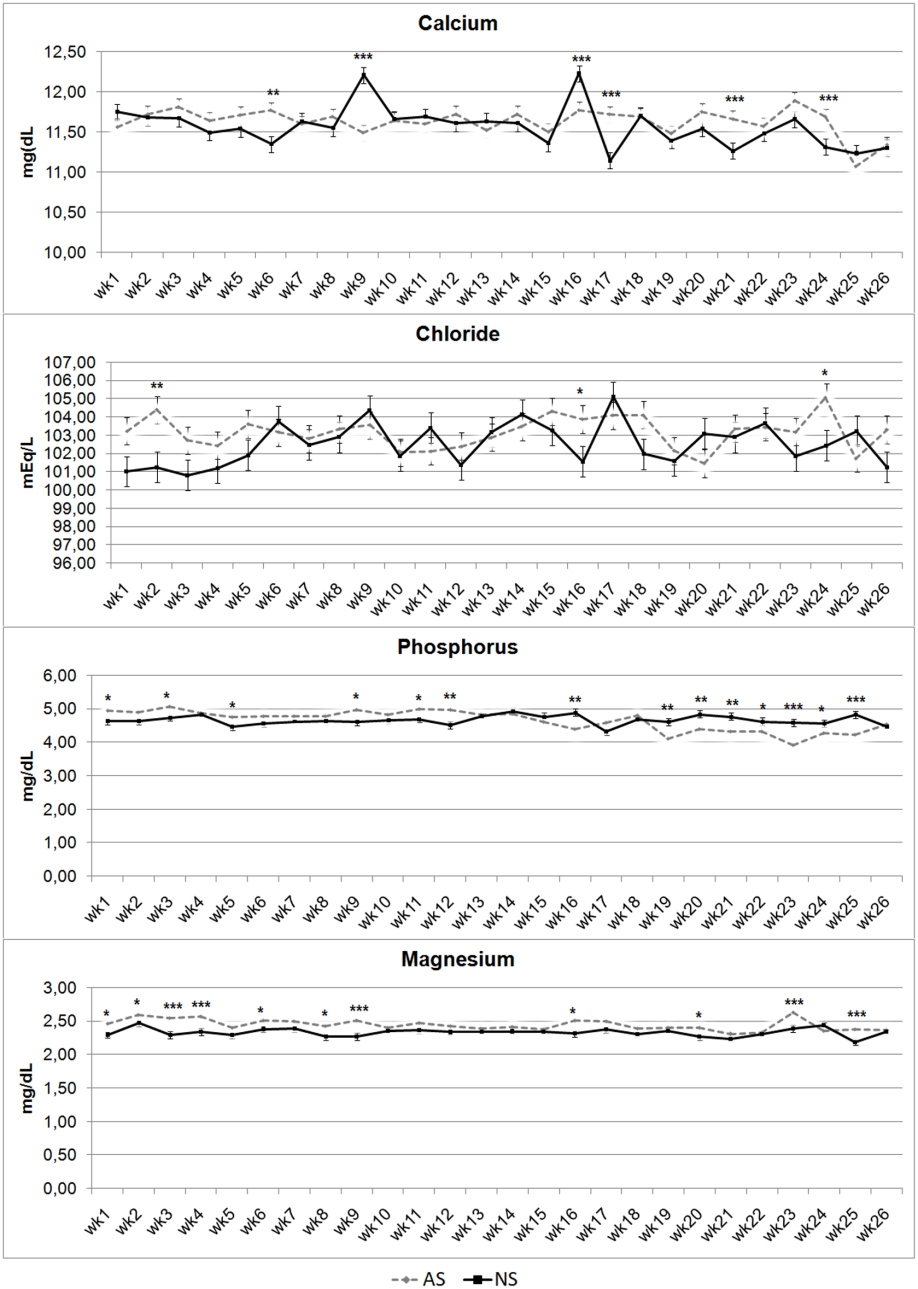
Calcium, chloride, phosphorous and magnesium serum concentrations in artificially-suckled (AS) and naturally-suckled (NS) donkey foals during the first 26 weeks of life. (Values are Least Square Means; bars represents Standard Error of the Mean). Statistical differences between the two groups at the same week were expressed: * = P < 0.05; ** = P < 0.01; *** = P < 0.001.

Fig 7 shows trends for blood mineral concentrations. Calcium concentration showed higher levels in AS foals at wks 6, 21, 24 (P < 0.01) and 17 (P < 0.001), while NS foal Ca levels showed greater concentrations at wks 9 (P < 0.001) and 16 (P < 0.01). Phosphate concentration had a different trend before and after wk 14–15. Particularly, P had higher levels in AS foals at wks 1, 3, 5,9, 11 (P < 0.05) and 12 (P < 0.01), with greater values in NS foals at wks 16. 19, 20, 21, 24 (P < 0.01), 22 (P < 0.05), 23 and 25 (P < 0.001). Cl showed higher concentrations in AS foals at wks 2 (P < 0.01), 16 and 24 (P < 0.05). Magnesium levels were higher in AS foals than in NS, specifically at wks 1, 2, 6, 8, 16, 20 (P < 0.05), 3, 4, 9, 23 and 25 (P < 0.001).

**Fig 8:**
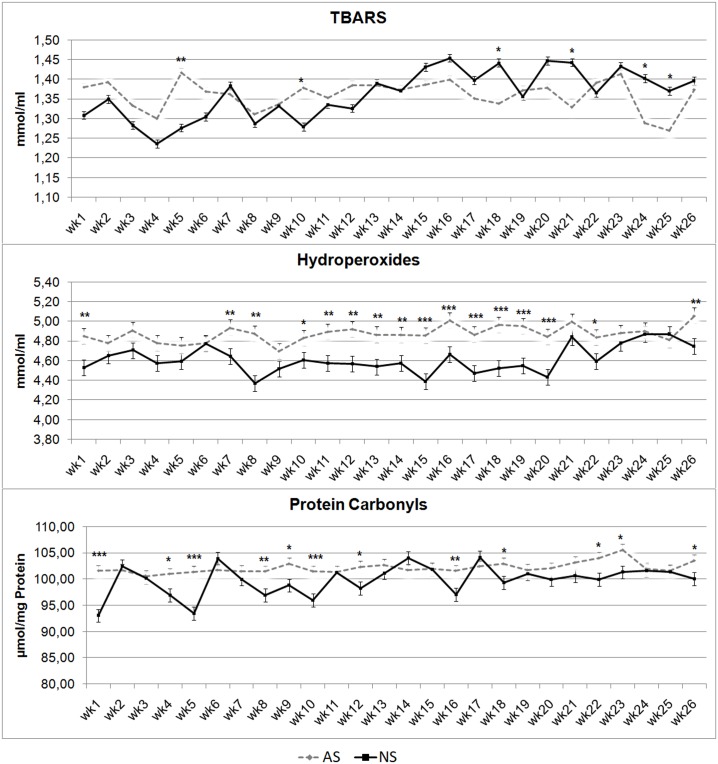
Thiobarbituric acid reactive substances (TBARS), hydroperoxides and protein carbonyls plasma concentrations in artificially-suckled (AS) and naturally-suckled (NS) donkey foalsduring the first 26 weeks of life (Values are Least Square Means; bars represents Standard Error of the Mean). Statistical differences between the two groups at the same week were expressed: * = P < 0.05; ** = P < 0.01; *** = P < 0.001.

Fig 8 shows the trends for oxidative biomarkers. The AS Group showed higher TBARS levels at wks 5 (P < 0.01) and 10 (P < 0.05). By contrast, at wks 18, 21, 24 and 25, TBARS of NS foals were higher (P < 0.05). Hydroperoxides were higher in AS than in NS foals throughout most of the experimental period, assuming statistical significance at wks 1,7, 11, 12, 13, 14, 26 (P < 0.01), 10 (P < 0.05),8, 15 to 20 (P < 0.001), 10 and 22 (P < 0.05). Protein carbonyl in AS group showed higher values at wks 1, 5, 10 (P < 0.001), 4, 9, 12, 18, 22, 26 (P < 0.05), 8, 16, and 23 (P < 0.01).

## Discussion

The overall data obtained from the serum biochemical profile in donkey foals showed ranges almost comparable with those reported in literature in donkey foals[13, 14, 15, 16]. Actually, the scientific literature available is rather poor compared with other farm animals. However, on comparing each recorded parameter with the same parameters reported in literature, some differences can be found which may be caused by multiple variables such as different techniques and/or instruments, breeds, local environments, variations in the time of blood sample collection for biochemical analytes subject to circadian rhythm [17, 18].

During the 12h of separation from dams, donkey foals in the NS group had an increasing consumption of solid feed during the experimental trial that would satisfy their nutritional requirements only at an age closer to weaning (starting from wk 10). Thus, the NS group had some weeks(at least the first two weeks) of effective fasting from 8.00 to 20.00. Therefore, the substantial difference between the AS and NS groups, from a metabolic point of view, is the availability of feed capable of satisfying nutritional needs during the 12h of separation from their dams. According to reference knowledge [19] on horse foals, fasting during the earlier phases of life could elicit metabolic and homeostatic disorders, but this was not apparent from our trial on donkey foals. On the other hand, because our blood sampling plan started at 7 days after birth, we cannot exclude that some subclinical metabolic alterations occurred during the foals’ first days of life. Few studies have investigated weekly variations in the biochemical profile during the first 26 weeks of life in donkey foals. Studies on the biochemical profile of newborn Martina Franca donkey foals have revealed many significant variations in several analytes in the first week of life, performing samples every day, and during the second and third weeks of life [16]. The onlypaper reporting a weekly blood sampling protocol in Amiata donkey foals from birth to 8 weeks is by [15]. Comparing our results with theirs confirmed the influence of age during the first weeks of life on AST, Glu and CPK. However,, we also found a strong influence of age in the first 26 wks of life on Crea, BUN, Trig, Chol, Ca, P and Alb. This may be due to the longer evaluation of the trends for these analytes in our paper than in others [15], but also to substantial differences in breed, management, environment, and analysis technique between the two trials. We have no reference data comparable with our results on the effect of age on LDH, NEFA, TP, Glob, AG, Cl and Mg. Few studies have investigated ALT and ALP in donkey foals[15, 16], both of which observed higher ALP values in the first days of donkey foal life, but they reach values similar to what we observed from the 2^nd^ week of life onwards. Although the values for each biochemical analyte are within reference intervals, many of them were influenced by suckling technique. This means that a fasting period of 12h per day or the availability of milk replacer strongly influenced the biochemical profile in donkey foals, without ever activating pathological pathways, so the main differences in biochemical profile are due to homeostatic mechanisms or to different development or activity of some organs.

The maximum difference between AST values was recorded, with statistically significant evidence, during wks 12 to 16. Differently, ALT showed higher differences than AST between AS and NS foals, with consistently higher concentrations in the AS group. Considering that ALT activity is related to liver function [20], it could be that AS foals had higher hepatic metabolic activity, or higher development, during the artificial-suckling period, probably due to digestive processes. To the best of the authors’ knowledge, this is the first study to measure NEFA values in donkey foals, so this analyte cannot be compared with results from other trials, but if compared with values in adult horses, NEFA showed similar concentrations[21]. Even though NS foals were fasted for 12h per day, they showed neither metabolic imbalance nor metabolic deficits. In fact, they show high resistance and resilience to short fasting periods, even repeatedly on a daily basis.

Alkaline phosphatase has widespread tissue distribution, with several isoenzymes isolated from liver, bone, intestinal mucosa, kidney and leucocytes[22]. The higher levels in the AS group could be explained by the greater bone and intestinal activity of AS foals. Indeed, during the 12h of separation from their dams, AS foals had the availability of milk replacer, thus increasing ALP values due to the metabolic activity of the small intestine mucosa, as they absorb carbohydrates [23]. Besides, De Palo et al. [5] showed that partially artificially-suckled donkey foals had higher growth rates than traditionally managed animals, so higher ALP levels could be linked to a hypothetically higher metabolic osteosynthetic activity.

Creatinine levels recorded in our trial showed superimposable values comparing to values reported for foals [15] and adult donkeys [24] whereas BUN levels were similar to those of foals [15] but lower than those recorded in adult donkeys [25]. We can thus conclude that both groups had no renal and/or muscular disorders caused by the feeding and management technique. Blood urea nitrogen variability is probably due to the influence that feeding management and ration characteristics (proteins availability and their metabolism) have on this analyte [26]. During the experimental period, both Crea and BUN showed some variation over time, that could be related respectively to muscular activity and to the different protein intake (due to the different feed intake over time) by foals.

Also of great interest are the results concerning energy profiles in foals: glucose remained constant throughout the experimental period for both groups, as reported for donkey foals [15] indicating that donkey foals have a strong homeostatic control of glucose in the blood even in the first weeks of life, probably due to this species’ high resistance to glucose metabolism disorders, but also because the fasting lasted no more than 12h, despite being repeated daily.

Some interesting variations are evident in lipid metabolism: from the first week of life until weeks 11–16 Trig values were higher in AS foals, a sign that their ration was more nutritious due to the intake of large amounts of milk replacer. Trig values are often studied in donkey to evaluate the pathological condition of hyperlipemia/hyperlipidemic syndrome strictly linked to a negative energy balance and/or fasting. In our trial, we found no blood biochemical signals of this pathological condition. Also in this case, the reason for the absence of this pre-clinical condition could lie in the short-term condition of fasting applied in NS animals, despite being repeated daily.

While Chol did not show any interesting trends in our trial, evaluating the results for NEFA we observed a tendency to higher levels for this analyte in NS foals, particularly in the first 11 weeks of life. We could hypothesize a tendency on the part of donkey species to satisfy their energy needs during short-term fasting periods by lipomobilization and emphasizing the lipid metabolism more than the carbohydrate one. Indeed, we found that NEFA are higher in NS group from wk1 to wk 23. Besides, in natural conditions, particularly during the first two weeks of life, donkey foals tend to suckle frequently, about every 15–20 min. Wild ass foals suckle every 3–10 min for the first 5 days, but by 10 days they suckle only every 20–30 min [27]. Therefore, given that in natural conditions donkey foals tend to suckle as frequently on milk that is so lactose-rich, a 12-hour fasting period may increase such metabolic pathways as glycogen lysis, and fat deposit mobilization. These aspects are in any case unclear and need to be further investigated.

Total protein tends to be higher in AS foals starting from wk 12until the second week after weaning (wk 26). Besides, it clearly emerges that while NS foals show high levels of Alb, AS foals reveal high Glob values. Differences reduce closer to the weaning period. This indicates that the suckling technique influences these parameters, reducing the differences after weaning. The increase in albumin is probably related to the development of the liver, its main production site [28]. This confirms that animals’ organ functions are in a development phase during the early neonatal period and a wide range of physiological values in different systems compensate for immaturity [29].

For Glob, results are difficult to discuss precisely, as their fractions tends to vary according to the analytical system, and the physiological role of each fraction is unknown [13]. In any case, of the Glob fractions, γ-globulins have the highest concentrations [30], so it can be presumed that AS foals tended to have higher antibody production, although data obtained by the preset trial are not able to justify this record. Of course, the A/G values confirm the different patterns of Alb and Glob in the two experimental groups of foals.

All of the minerals investigated showed a constant trend throughout the experimental period, and all the values recorded are similar to those reported in the literature for donkey foals [15]. This leads to the conclusion that the mineral profile is also not influenced by suckling technique and that Martina Franca donkey foals have a high efficiency in maintaining blood mineral homeostasis.

The present paper shows, for the first time, to the best of our knowledge, results outlining some oxidative biomarkers in donkey: TBARS, PC and Hy. While TBARS and Hy are related to lipid oxidation, PC is an analyte that highlights protein oxidation [31]. These parameters have been investigated in horse blood plasma, where almost all studies and reviews pertained to exercise-induced oxidative stress [32, 33]. Comparing our results on these analytes with those recorded in adult horses, we found lower concentrations of TBARS, Hy and PC [31, 34]. Evaluating the trend of these oxidative markers in blood plasma during the experimental trial in the AS and NS groups highlighted that TBARS and PC were unchanged during the trial in both groups, highlighting that no increasing in oxidative metabolic pathways in foals occurred in the two experimental groups during the trial period. A statistically significant higher value tendency in Hy was recorded in AS than in NS. This could be due to the different lipid metabolism showed by AS foals to NS foals, as confirmed by the trends for Trig and NEFA.

Several authors recorded a nonspecific reactivity of TBA that clearly renders the test poorly suitable for determination of lipid hydroperoxides and MDA in body fluids [35]. Although this technique presents these limitations, the results obtained in the present work are scientifically sound in terms of comparison between the two experimental groups, as we used the same techniques for sampling and laboratory analysis.

## Conclusions

This paper shows that suckling technique affects biochemical profiles and lipid peroxidation patterns in donkey foals. Besides, we found that separating foals from their dams for 12 hours daily for 24 weeks brings animals’ biochemical parameters within the reference intervals. This highlights, at least from a metabolic point of view and exclusively for the parameters investigated, that these are healthy conditions for donkey foals. But these are not necessarily the best conditions for high milk production.

## Supporting information

S1 Table Composition of starter, oat hay and milk replacer supplied to donkeys. (DOCX)

S2 Table Blood parameters, analytical methods, and quality laboratory assays. (DOCX)
