# Supplementary material for: Survey of biochemical and oxidative profile in donkey foals suckled with one natural and one semi-artificial technique
Source: PLoS One. 2018 Jun 6;13(6):e0198774. doi: 10.1371/journal.pone.0198774 (PMC5991377; doi:10.1371/journal.pone.0198774)
Supplement: S1 Table — (DOCX) [file pone.0198774.s001.docx]

*Composition of starter, oat hay and milk replacer supplied to donkeys*

|  | Starter^1^ | Oat hay | Milk replacer^2^ |
| --- | --- | --- | --- |
| DM | 86.5% | 88.8 | - |
| Protein | 17.1% | 11.4 | 26 % |
| Fat | 5.76% | 2.75 | 22 % |
| Fiber | 10.4% | 34.2 | 0.05 % |
| Ash | 6.8% | 10.8 | 6.5 % |
| Neutral detergent fibre | 27.7% | 55.4 | - |
| Acid detergent fibre | 13.4% | 38.9 | - |
| Acid detergent lignin | 2.6% | 7.1 | - |
| Horse forage units, n/kg of DM | 0.85% | 0.48 | - |
| Digestible protein, g/kg | 108.4% | 20.4 | - |
| Sodium | - | - | 0.7 % |
| Vitamin A | - | - | 50 000 IU/kg |
| Vitamin D_3_ | - | - | 5 000 IU/kg |
| Vitamin E | - | - | 100 mg/kg |
| Vitamin C | - | - | 100 mg/kg |
| Iron (iron (II) sulphate) | - | - | 80 mg/kg |
| Zinc (zinc sulphate) | - | - | 70 mg/kg |
| Manganese (manganese sulphate) | - | - | 55 mg/kg |
| Copper (copper (II) sulphate) | - | - | 8 mg/kg |
| Iodine (calcium iodate) | - | - | 1 mg/kg |
| Selenium (sodium selenate) | - | - | 0.25 mg/kg |

^1^Composition: Rolled corn 33%, soybean meal 21%, wheat bran 17%, rolled barley 13.5%, rolled oats 13.5%, vitamin and mineral integration 2%).

^2^Composition: cow milk serum protein concentrate, whey without lactose, sweet whey, coconut oil, palm oil, wheat protein concentrate, wheat flour
