# Supplementary material for: Survey of biochemical and oxidative profile in donkey foals suckled with one natural and one semi-artificial technique
Source: PLoS One. 2018 Jun 6;13(6):e0198774. doi: 10.1371/journal.pone.0198774 (PMC5991377; doi:10.1371/journal.pone.0198774)
Supplement: S2 Table — (DOCX) [file pone.0198774.s002.docx]

| **Parameter^1^** | **Analytical method** | **Intra-assay variation (CV%)** | **Inter-assay variation (CV%)** |
| --- | --- | --- | --- |
| Total protein | Biuret method | 0.75 | 0.85 |
| Albumin | Colorimetric bromocresol green (BCG) method | 1.29 | 2.09 |
| Urea | UV kinetic (urease and glutamate dehydrogenase GLDH) | 1.89 | 2.99 |
| Creatinine | Jaffè colorimetric method without deproteinization | 1.34 | 2.21 |
| Glucose | Colorimetric enzymatic method GOD-POD | 1.21 | 1.08 |
| NEFA | Colorimetric method | 1.78 | 1.41 |
| Triglyceride | Colorimetric enzymatic method (GPO)-PAP | 3.69 | 4.05 |
| ALT | Kinetic UV Optimized method IFCC | 4.37 | 4.52 |
| AST | Kinetic UV Optimized method IFCC | 1.12 | 1.32 |
| ALP | Colorimetric Optimized method DEA DG KC | 0.49 | 0.57 |
| LDH | Kinetic method | 2.04 | 2.35 |
| CK | Enzymatic method | 1.09 | 1.36 |
| Cholesterol | Colorimetric Enzymatic method (CHOD)-PAP | 2.11 | 2.86 |
| Calcium | Colorimetric arsenazo method | 0.96 | 0.63 |
| Phosphorus | Colorimetric blue of molybdate method | 0.77 | 1.26 |
| Magnesium | Colorimetric Xylidyl blue method | 1.26 | 2.90 |
| Chlorine | Ion selective electrode method | 0.31 | 1.02 |
| TBARs | Spectrophotometric method | 1.86 | 2.15 |
| Lipid hydroperoxides | Spectrophotometric method | 1.49 | 2.41 |
| Protein carbonyls | Spectrophotometric method | 1.66 | 2.33 |

Blood parameters, analytical methods, and quality laboratory assays

^1^ALT = alanine aminotransferase; AST = aspartate aminotransferase; ALP = alkaline phosphatase; LDH = lactate dehydrogenase; CPK = creatinine phosphokinase; TBARs: Thiobarbituric acid reactive substances.
